# Quorum sensing in human gut and food microbiomes: Significance and potential for therapeutic targeting

**DOI:** 10.3389/fmicb.2022.1002185

**Published:** 2022-11-25

**Authors:** A. Kate Falà, Avelino Álvarez-Ordóñez, Alain Filloux, Cormac G. M. Gahan, Paul D. Cotter

**Affiliations:** ^1^APC Microbiome Ireland, University College Cork, Cork, Ireland; ^2^School of Microbiology, University College Cork, Cork, Ireland; ^3^Food Bioscience Department, Teagasc Food Research Centre, Fermoy, Ireland; ^4^Department of Food Hygiene and Technology and Institute of Food Science and Technology, Universidad de León, León, Spain; ^5^MRC Centre for Molecular Bacteriology and Infection, Department of Life Sciences, Imperial College London, London, United Kingdom; ^6^School of Pharmacy, University College Cork, Cork, Ireland

**Keywords:** quorum sensing, quorum quenching, quorum sensing inhibition, gut microbiome, food microbiome, food matrix

## Abstract

Human gut and food microbiomes interact during digestion. The outcome of these interactions influences the taxonomical composition and functional capacity of the resident human gut microbiome, with potential consequential impacts on health and disease. Microbe-microbe interactions between the resident and introduced microbiomes, which likely influence host colonisation, are orchestrated by environmental conditions, elements of the food matrix, host-associated factors as well as social cues from other microorganisms. Quorum sensing is one example of a social cue that allows bacterial communities to regulate genetic expression based on their respective population density and has emerged as an attractive target for therapeutic intervention. By interfering with bacterial quorum sensing, for instance, enzymatic degradation of signalling molecules (quorum quenching) or the application of quorum sensing inhibitory compounds, it may be possible to modulate the microbial composition of communities of interest without incurring negative effects associated with traditional antimicrobial approaches. In this review, we summarise and critically discuss the literature relating to quorum sensing from the perspective of the interactions between the food and human gut microbiome, providing a general overview of the current understanding of the prevalence and influence of quorum sensing in this context, and assessing the potential for therapeutic targeting of quorum sensing mechanisms.

## Introduction

The interactions between human, animal and plant microbiomes and their ultimate impact on the assembly and maintenance of community structure and functionality is the focus of intense research efforts (Jagadeesan et al., 2019). In food microbiology, the influence of ingested food microbiomes on the human gut is a particular focus in recent years. The human gut harbours the greatest microbial load of all body sites − estimated to be in the order of 10^13^ bacterial cells (along with eukaryotic microorganisms and phage; Sender et al., 2016). This resident microbiota plays a significant role in human health and is amenable to outside influences imposed by diet and organisms that are resident in raw and minimally processed foods (Janssens et al., 2018; Walter et al., 2020). Nevertheless, many knowledge gaps persist regarding the dynamics of colonisation, resistance and succession in the food and gut microbiomes and how these may be modulated.

The genetic repertoire of bacteria enables them to perceive and adapt to environmental factors in flux. Environmental sensing and signalling in bacteria is primarily achieved through secondary nucleotide messengers, such as cyclic adenosine monophosphate (cAMP), cyclic diguanylate (c-di-GMP) and the hyperphosphorylated guanosine derivatives collectively referred to as (p)ppGpp (Hengge et al., 2019), as well as Two-Component Systems (TCSs) − pairs of sensory histidine kinases and response regulators that perceive extracellular signals and modulate gene expression accordingly (Liu et al., 2018a). In addition, many bacterial species possess dedicated Quorum Sensing (QS) systems that involve the secretion, extracellular accumulation and subsequent import and processing of specific small chemical signal molecules (Eickhoff and Bassler, 2018). At threshold concentrations, these signal molecules in the extracellular environment induce transcriptional changes that trigger phenotypic adaptation to changing social contexts. These include the coordination of biofilm regulation, expression of virulence or food spoilage traits and social choices such as cooperation, competition and cheating within micro-ecological contexts (Li and Tian, 2012; Allen et al., 2016; Defoirdt, 2018).

QS systems can provide information on the local population of ‘self’ and ‘other’; and it has been also proposed that QS could enable bacteria to detect diffusion-limited situations through an integrated model of efficiency sensing (Hense et al., 2007; Liu et al., 2018a). QS systems are described in both Gram-negative (Papenfort and Bassler, 2016) and Gram-positive bacteria (Verbeke et al., 2017), as well as fungi (Nogueira et al., 2019) and some viruses (Silpe and Bassler, 2019). Furthermore, next-generation sequencing and comparative genomic approaches have identified homologues of known quorum sensing circuits across genus and species boundaries (Whiteley et al., 2017), suggesting its importance in the regulation of survival strategies.

This review summarises the fundamental and translational research to date relating to the targeting of bacterial quorum sensing to produce foods with enhanced safety and quality profiles and the modulation of gut microbiomes for human health. An updated catalogue of quorum sensing activity and its inhibition in foods and the human gut is provided. Finally, we identify and discuss gaps in the literature that can be addressed in order to progress research in this field specifically with a view to the bioprotection of foods and the therapeutic targeting of the gut microbiome. As this review demonstrates, there is great promise for direct applications in food production systems, particularly in the context of functional foods, as well as biotherapeutics in the clinical sphere. On the other hand, some of the gaps identified within have the potential, if addressed, to benefit broader research in the field of microbial ecology in a cross-disciplinary manner.

## Quorum sensing in bacteria

**Figure 1:**
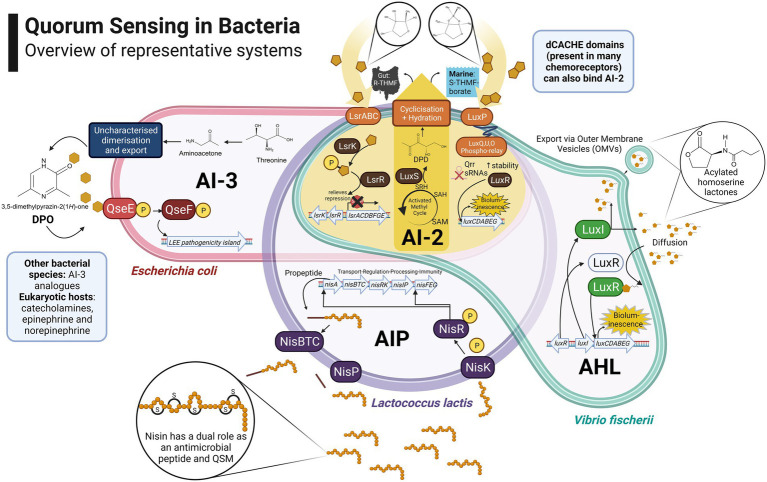
Overview of representative bacterial quorum sensing systems: *Escherichia coli* cell illustrates an Autoinducer 3 (AI-3) Quorum Sensing (QS) system which regulates the locus of enterocyte effacement (LEE) pathogenicity island (Walters and Sperandio, 2006; Machado Ribeiro et al., 2021); an Autoinducing Peptide (AIP) system in *Lactococcus lactis* is presented, in which nisin has a dual role as a Quorum Sensing Molecule (QSM) and antimicrobial peptide (Kleerebezem, 2004); *Vibrio fischeri* cell illustrates the control of bioluminescence through an Acylated Homoserine Lactone (AHL) system also referred to as Autoinducer 1 (AI-1; Nealson, Platt and Woodland Hastings, 1970); Autoinducer 2 (AI-2) signalling is presented at the intersection due to its role in species- and genera-agnostic communications. The AI-2 biosynthetic pathway is highlighted in yellow alongside the *Lsr/Lux* pathways which provide for its internalisation/detection in various genera (Pereira, Thompson and Xavier, 2013). Created with BioRender.com.

As per the original system first identified in *Vibrio fischeri*, the simplest QS circuit consists of regulatory gene pairs responsible for the production of signalling molecules (*luxI*) and transcriptional activation (*luxR*) of regulated genes responsible for phenotypes such as bioluminescence (Nealson et al., 1970). In addition to global phenotypic regulation based on local population density, QS can also contribute to managing the production of public goods including both the signal molecules themselves (auto-induction) and co-regulated products relevant to the context of foods and the human gut, such as extracellular enzymes, exopolysaccharides, surfactants, antimicrobial compounds, virulence factors and siderophores (Heilmann et al., 2015). An overview of the principal systems found in bacteria is presented in Figure 1. Public goods, in the context of microbiology, refers to molecular components of the secretome produced by individual cells and transferred to the extracellular milieu, potentially benefiting the population as a whole. Public goods are distinct from private goods, which are generally cytosolic or membrane-bound and hence confer an exclusive benefit upon producer cells.

Understanding the relative contribution of quorum sensing to microbiome structure and functionality is complicated by heterogeneous distribution of QS producers and responders, even within clonal populations (Striednig and Hilbi, 2022). For instance, one study reported that just 68% of *V. fischeri* cells responded to exogenous QSM (Pérez and Hagen, 2010). Further, receptor promiscuity and divergence in ligand binding of quorum sensing receptors towards non-canonical substrates is suggestive of a system that can be tuned and can adapt over time to changing environmental conditions and competitors (Hawver et al., 2016; Prescott and Decho, 2020). Mapping QS systems in complex ecological environments is complicated by their tendency to follow hierarchical and combinatorial structures (Ng and Bassler, 2009; Cornforth et al., 2014) and to modulate gene expression programs through asymmetry (Hurley and Bassler, 2017), thought to facilitate the management of metabolic trade-offs (Dinh and Prather, 2019). This is perhaps best exemplified by the Qrr sRNAs (Quorum Regulatory sRNAs) of the *Vibrio* genus and the multifunctional RNAIII which coordinates several stacked regulatory circuits managing the accessory gene regulator (*agr*) system in *Staphylococcus aureus* (Bronesky et al., 2016).

### Quorum sensing molecules

QS systems involve the production and detection of Quorum Sensing Molecules (QSM) and understanding the synthesis, structure and stability of these compounds is important for developing strategies for therapeutic targeting of these systems. A high-level overview of the principal classes of QSM is provided in Table 1.

**Table 1:** Overview of major bacterial Quorum Sensing Molecules (QSM) families, examples, possible modifications, producer and/or responder bacteria utilising the QSM as well as the regulated phenotypes and specificity.

System	QSM	QSM modification	Producer (and responder) bacteria	Responder bacteria (no production)	Relevant phenotypes regulated	Specificity	Reference
Autoinducer 1 (AI-1)	Acylated-homoserine lactones (AHL/HSL)	Homoserine lactone moiety is joined to a variable length (C4-C16) acyl tail, which can bear oxo- or hydroxyl- substitutions.	Gram-negative bacteria, e.g. *Aliivibrio fischeri*	Species with LuxR solos	*las* and *rhl* systems	Generally species-specific due to AHL molecular structure (length of acyl chain; substitutions)	Fuqua, Parsek and Greenberg, (2001); Bez et al., (2021)
Autoinducer 2 (AI-2)	*S*-THMF (*2S*,*4S*)-2-methyl-2,3,3,4-tetra-hydroxytetrahydrofuran; *R*-THMF: (*2R*,*4S*)-2-methyl-2,3,3,4-tetrahydroxytetrahydro-furan)	*S*-THMF-borate predominates in marine environments; heptyl modifications are possible	~Half of sequenced bacterial genomes through *luxS*	*Pseudomonas aeruginosa*, *Bacillus subtilis* and *Rhodopseudomonas palustris* (through dCACHE domain)	Multiple and varied	Species-agnostic; enteric bacteria detect *R*-THMF through *lsr* operon; boronated *S*-THMF is detected through *lux* cascade; other bacteria detect through dCACHE domain-containing proteins	Pereira, Thompson and Xavier, (2013)
Autoinducer 3 (AI-3)	3,5-dimethylpyrazin-2-one (DPO)	Various pyrazinone analogues	Enterohemorrhagic *Escherichia coli* (EHEC)	Many Gram-negative bacteria (through QseC homologues)	Expression of locus of enterocyte effacement (LEE), Shiga-toxin production	Interspecific (differences in relative abundance of AI-3 analogues); Inter-kingdom crosstalk with mammalian hormones epinephrine and norepinephrine.	Clarke et al., (2006); Walters and Sperandio, (2006); Kim et al., (2020); Machado Ribeiro et al., (2021)
Autoinducer peptides (AIP)	Post-translationally modified peptides	Length (5–17 amino acids); Linear or cyclical structure	Gram-positive bacteria, e.g. *Lactococcus lactis*		Sporulation and competence in *Bacillus subtilis* (*phr* system); toxin production in *C. botulinum* and *C. perfringens* (*agr*-type system)	Generally species-specific due to the molecular structure	Kleerebezem et al., (1997); Novick and Muir, (1999); Cook and Federle, (2014); Monnet, Juillard and Gardan, (2016)
Small RNAs	miRNAs and siRNAs; Qrr sRNAs (Quorum Regulatory sRNAs) in *Vibrio* spp.; RNAIII in *Staphylococcus aureus*	Specificity to targeted mRNA transcripts, which are removed from circulation.	Reported in *Escherichia*, *Salmonella*, *Streptococcus*, *Pseudomonas* and *Vibrio* genera		Type III secretion genes in *Vibrio harveyi*	Specificity to the targeted mRNA transcript	Rutherford et al., (2011); Teng et al., (2011); Fu, Elena and Marquez, (2019)
Diffusible Signal Factor (DSF)	*cis*-11-methyl-dodecenoic acid Derived from fatty acids with a cis-unsaturated double bond at the 2-position.	Chain length, branched/unbranched structure	*Stenotrophomonas maltophil*ia, *Burkholderia cenocepacia* and *P. aeruginosa*	*S. aureus, B. cereus, S. enterica, E. coli*	Virulence and exopolysaccharide (EPS) production in *Xanthomonas oryza*e pv. oryzae; Biofilm dispersal in *P. aeruginosa*	Intraspecific and cross-kingdom crosstalk possible	Sepehr et al., (2014); Zhou et al., (2017); Liu et al., (2018b)
Palmitate methyl ester (PAME)	3-hydroxy palmitic acid methyl ester		*Ralstonia solanacearum*		Virulence and upregulation of AHL production	Species-specific	Flavier et al., (1997); Mole et al., (2007)
Diketopiperazines (DKP)	Amino acid-derived cyclic dipeptides	Constituent amino acids; Chirality;	*Cronobacter sakazakii*, *Bacillus cereus*, *Vibrio* spp.		Biofilm formation in *C. sakazakii*; Increased resistance to oxidative stress in *Vibrio* spp.	cyclo(Phe-Pro) is detected by both *C. sakazakii* and *B. cereus*	Bofinger et al., (2017); Kim et al., (2018); Zink et al., (2021)
Quinolones	2-heptyl-3-hydroxy-4-quinolone (PQS); 2-alkyl-4(1H)-quinolone (AHQ)		*P. aeruginosa*		Virulence factors, including pyocyanin, elastase, lectin, and rhamnolipid production	Species-specific	Rampioni et al., (2016); Lin et al., (2018)
Dialkylresorcinols	Dialkylresorcinols (DARs) and Cyclohexanediones (CHDs)	Variable R chain	116 species, including the genera *Photorhabdus, Neisseria, Capnocytophaga, Flavobacterium*		Pathogenicity against *Galleria mellonella*	Intraspecific crosstalk possible	Brameyer et al., (2015)
Photopyrones (PPYs)	α-pyrones	Variable R chain	*Photorhabdus luminescens*	*Bacillus atrophaeus*	*Photorhabdus* clumping factor (Pcf); biofilm formation	Intraspecific crosstalk possible	Brachmann et al., (2013); Hickey et al., (2021)

There are many instances in which QSM fulfil a dual role in addition to QS, such as Autoinducer 2 (AI-2) and Autoinducer Peptides (AIPs). AI-2 systems have been identified in approximately half of sequenced bacterial genomes and are considered to mediate species-agnostic communication, particularly in complex communities, presented at the intersection of the bacterial cells in Figure 1. At the same time, some argue that AI-2 biosynthesis mainly contributes to the detoxification of S-adenosylhomocysteine (SAH) produced during the activated methyl cycle (AMC) and is essential to the metabolism of cysteine and methionine, with an incidental QS role (Kang et al., 2019). Disambiguation of these contrasting theories has been explored through the construction of a LuxS-deficient *Streptococcus mutans*, followed by restoration of AMC functionality by complementation with S-adenosyl-L-homocysteine hydrolase (*sahH* from *P. aeruginosa*; Hu et al., 2018). Whilst biofilm formation and acid tolerance were restored through complementation, homocysteine and lactic acid levels remained aberrant, underlining the broader significance of *luxS* in the metabolic landscape of the cell.

Similarly, there are numerous examples of AIP QSM which, in addition to facilitating QS, act as antimicrobial peptides such as lantibiotics (bacteriocins are reviewed in greater detail in Section 2.3). In addition to the multifunctionality of QSM themselves, it bears considering that QSM can also be degraded or for instance exported in a non-specific manner through multidrug efflux pumps (Rahmati et al., 2002).

### Methods for the analysis of QSM

As raised almost a decade ago (Skandamis and Nychas, 2012), a greater understanding of the relative abundance and significance of QS signalling in microbiomes is required, particularly in providing temporal resolution of QS at significant points in time and space (Donaldson et al., 2016). Accurate and rapid detection, characterisation and quantification of QSM can enhance our understanding of QS in various environments, as well as providing a real-time readout of microbial physiology within a given microbiome.

Conventional methods frequently used to interrogate the physico-chemical properties of QSM include mass spectrometry (MS) and high-performance liquid chromatography (HPLC), whilst biosensor-based systems employing plasmids, chromosomes or enzymes from reporter bacterial strains can rapidly detect a colorimetric, luminescent or fluorescent signal. Chromatographic techniques are more resource-intensive but can detect QSM with a higher degree of accuracy and can also be used to validate novel biosensors (Cutignano, 2019).

Whilst several analytical techniques are available for AI-1 and AI-2 QSM, AIP are generally more difficult to quantify as they are normally found at low concentrations in complex mixtures (Kalkum et al., 2003) whilst AI-3 was only recently characterised and no methods have been developed for its routine analysis yet (Kim et al., 2020). Novel approaches for the detection and characterisation of AIP and AI-3 are needed, in addition to protocols for QSM analysis in the complex matrices associated with food and human gut microbiome samples. In this regard, analytical techniques developed and validated in the context of environmental microbiology, such as the analysis of biofilms in wastewater treatment plants (Wang et al., 2018) could be adapted. These could be combined with emerging fluorescence-based techniques such as Combinatorial Labelling And Spectral Imaging-Fluorescence *In-Situ* Hybridization (CLASI-FISH) to incorporate the spatial ecology of microbial communities (Wilbert et al., 2020).

Several biosensor strains, often plasmid-based, have been constructed to detect different QSM classes (Wen et al., 2017), however chromatographic methods tend to be preferred to detect mixes of QSM in complex microbial consortia (Gui et al., 2018). Rapid paper-based diagnostic tools containing immobilised whole cell biosensors have also been validated to nanomolar level detection of AHLs and AI-2 QSM (Wynn et al., 2018).

Biochemical analytical techniques are increasingly being paired with *in silico* studies of quorum sensing-related genes in metagenomic samples to help distinguish QS potential from active QS processes (Rezzonico et al., 2012). In addition to the established Quorumpeps database for AIP peptides (Wynendaele et al., 2013), more recent efforts have generated novel databases including the Omics Database of Fermentative Microbes (ODFM; Whon et al., 2021) and Quorum Sensing of Human Gut Microbes (QSHGM; Wu et al., 2021b). Curated and regularly updated databases are fundamental for accurate bioinformatic predictions, which will assist in modelling the effect of quorum sensing systems in complex communities as part of broader pipelines (Wynendaele et al., 2013). Accurate modelling of quorum sensing in foods and the human gut is necessary in order to establish prevalence, relevance and to evaluate potential interventions. *Ex vivo* modelling of the human gut, such as through colonic models and gut-on-a-chip approaches, can provide an opportunity to benchmark quorum sensing activity at various points in the digestive process (Garcia-Gutierrez and Cotter, 2021). Fermented foods have also been demonstrated to be tractable and convenient experimental models for studying community assemblage in food microbiomes (Wolfe and Dutton, 2015) and could be evaluated as models for QS studies.

### Quorum sensing “eavesdropping”

Whilst QS was originally described in terms of autoinduction, communication through QS can occur across strains, species and even kingdoms (Kendall and Sperandio, 2016). LuxR solos are one such circuit that permit ‘eavesdropping’ by detecting and integrating community-level information to inform the regulation of gene expression without investing in the production of QSM (Subramoni and Venturi, 2009). The metabolic cost of QSM synthesis varies considerably – estimated in the order of 184 ATP per autoinducer peptide (AIP), 8 ATP per acyl-homoserine lactone (AHL) and 0–1 ATP per autoinducer-2 moiety (AI-2; Ruparell et al., 2016), highlighting the likely evolutionary benefit of an eavesdropping strategy.

Notable examples in an enteric context include species of the genera *Escherichia*, *Klebsiella*, and *Salmonella* that possess orthologues of the transcriptional regulatory protein SdiA − detecting AHLs produced by other species – whilst lacking the requisite genes for AHL production (Michael et al., 2001; Lindsay and Ahmer, 2005). This type of QS circuit is implicated in the suppression of *traI*-mediated conjugation between *E. coli* and *P. aeruginosa* (Lu et al., 2017), which could potentially be harnessed in applications to reduce dissemination of genetic determinants of antimicrobial resistance (AMR) by horizontal gene transfer (HGT). Similarly, several instances of food spoilage appear to be augmented by synergy between AHL-QSM producers and non-producers, presumably due to the capacity of the non-producers to detect QSM in the extracellular milieu (Yu et al., 2019; Wang and Xie, 2020).

### Quorum sensing inhibition

Due to the wide range of bacterial phenotypes regulated by quorum sensing and their impact in clinical, environmental and agricultural contexts, QS systems have been proposed as a potential therapeutic target for the modulation of microbial communities (Rutherford and Bassler, 2012; Kalia, 2013; Lade et al., 2014). Indeed, the phenomenon of quorum sensing inhibition (QSI; also referred to in the literature as quorum sensing interference to describe broader modulation of QS) has been reported not just amongst bacteria, suggesting an ecological role in governance of cooperation and competition (Murugayah and Gerth, 2019), but also between bacteria and eukaryotic cells including fungi, plants and animals (Hughes and Sperandio, 2008; Joshi et al., 2021). QSI can be accomplished through several mechanisms, such as enzymatic degradation [also referred to as Quorum Quenching (QQ)], production of QSM analogues, signal sequestration or other modulation of signal flux up or downstream.

Whilst various applications in medical and industrial settings have been investigated, the use of QSI to modulate food microbiomes and gut microbiota has received relatively less attention, yet may prove useful in addressing the global threat of antibiotic resistance and enhancing the robustness of probiotic strains (Kareb and Aïder, 2020). It is anticipated that metagenomics and metatranscriptomics will guide studies in this relatively novel area to determine the effect of QSI on microbial communities, whilst genetic engineering can be used to improve and maximise the potential of QQ enzymes derived from natural environments (Billot et al., 2020). Advances in the field of chemistry such as structure–activity studies of synthetic QSM analogues (Rajamani et al., 2008; Lyons et al., 2020) naturally complement such bioprospecting and rational design approaches.

Perhaps some of the most exciting research in the field has emerged at the interdisciplinary juncture between biology and engineering occupied by the field of synthetic biology (Wang et al., 2020). Given the close resemblance between electronic circuits and the intrinsic networks and feedback loops of quorum sensing systems, there is potential for ground-breaking interdisciplinary work. In the field of synthetic ecology, precisely engineered consortia are being used to evaluate the relative importance of quorum sensing pathways and the equilibrium between QS and QSI when targeted for therapeutic modulation (Stephens and Bentley, 2020), as well as advancing the fundamental research in the field (Wu et al., 2021a).

### Biofilms as environments which favour QS

Biofilms are aggregations of microbial cells, attached to one another and typically adhered to a surface through a matrix of polymers that are at least partially microbially secreted. They are ubiquitous and considered the predominant form of living adopted by bacteria, fungi, viruses and single-celled eukaryotes (Flemming et al., 2016), with specific relevance to food and food processing environments (Alvarez-Ordóñez et al., 2019) and the human gut (Deng et al., 2020).

As well as physically shielding in-dwelling cells, the structure of the biofilm matrix includes channels and microcolonies that generate nutrient and oxygen gradients. These gradients are thought to contribute to phenotypic and genotypic plasticity and reinforce the heterogeneity generally seen in biofilm-dwelling populations (Penesyan et al., 2019). The distinct microenvironments formed and defined by biofilms are thought to favour interactions with other microorganisms due to their enforced proximity within the matrix and reduced diffusion. The resulting circulation of nutrients, metabolites, QSM and other public goods such as extracellular enzymes and siderophores are considered to influence emergent features in bacteria (Solano et al., 2014; Flemming et al., 2016). Emergent properties refer to characteristics of populations, especially multispecies and biofilm-associated, which differ from those of equivalent planktonic populations. Most commonly studied attributes include social cooperation, nutrient management and antimicrobial tolerance (Flemming et al., 2016).

More specifically, mixed biofilms containing multiple species are commonplace. They may arise due to synergy between different attachment phenotypes, such as specialised fimbriae and exopolysaccharide (EPS) production, with the structure of the latter in particular being influential on the emergent properties of biofilms beyond those of equivalent planktonic populations (Swaggerty et al., 2018).

## Quorum sensing in foods and food processing environments

Advances in nucleic acid manipulation and sequencing have helped enhance our understanding of food microbiomes: the microbial communities within and upon food matrices, which become established during primary production and evolve throughout the farm-to-fork continuum (De Filippis et al., 2018; Yap et al., 2022). This field of study receives special attention in light of the emerging, cross-disciplinary approach of One Health, which recognises the interrelationship between human, animal and environmental health [One Health High-Level Expert Panel (OHHLEP) et al., 2022]. These advances have helped the collection of temporal datasets that can better describe the taxonomical and functional evolution of microbiomes across complex food chains, in order to enhance our understanding of their influence on food safety, quality, sustainability and potential impact on human health (Pasolli et al., 2020). Whilst certain species can dominate specific communities at particular times, broadly speaking food microbiomes consist of multiple strains with some degree of interaction, which may affect collective behaviours of microorganisms such as food spoilage (Qian et al., 2018). In regulating phenotypic characteristics and metabolic processes, QS is proposed to enhance microbial adaptation to dynamic environments and has been associated with food quality in both a negative sense with respect to microbial food spoilage (Yuan et al., 2018), as well as enhancing the quality of some fermented foods (Johansen and Jespersen, 2017).

The overall impact of QS in microbial succession dynamics within food microbiomes is difficult to assess due to the frequent heterogeneous responses seen in bacteria to QSM described previously. Further, relative receptor binding affinity studies have demonstrated that many QS receptors can be activated by many other small molecules produced by microorganisms or hosts (Wellington and Greenberg, 2019), as we will outline for various compounds present in foods. Finally, the accumulation, compartmentalisation and stability of QSM are likely to be highly influenced by intrinsic and extrinsic factors of the food matrix and subject to fluctuations at different points of the food production process (Skandamis and Nychas, 2012). For example, studies on AHL-QSM stability in soil established a half-life ranging from hours to days depending on pH and temperature (Delalande, 2005) and the influence of micro-environmental conditions in foods and the human gut remains to be elucidated. Recent technologies have harnessed bacterial QS activity to provide real-time monitoring of microbial activity during food production (Miller and Gilmore, 2020).

### Quorum sensing molecules detected in foods

**Figure 2:**
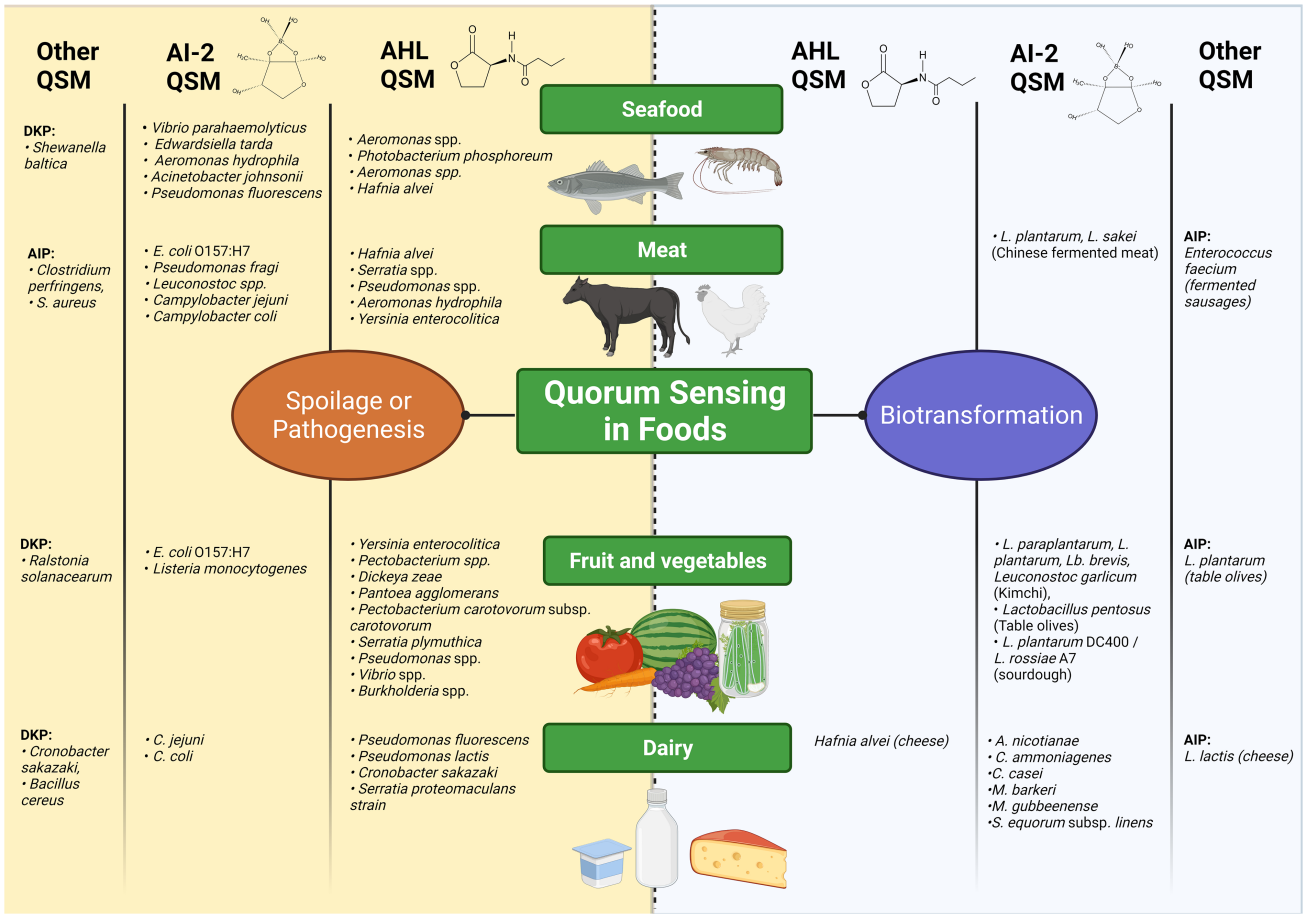
Overview of Quorum Sensing Processes as involved in food production, adapted from (Blana, Doulgeraki and Nychas, 2011; Plummer, 2012; Nahar et al., 2018; Quintieri et al., 2020; Wang and Xie, 2020). The left hand side of the figure in yellow maps food-associated bacteria reported in the literature to utilise QS for spoilage or pathogenesis, organised into columns based on the class of QSM (AHL, AI-2 and others) and by the type of food in which they are reported. This scheme is repeated on the right hand side in blue for those food-borne bacteria with positive attributes in food with respect to biopreservation and biotransformation. Created with BioRender.com.

A broad range of QSM have been reported in different foods, as illustrated in Figure 2. Overall, fatty acid-derived QSM such as AHLs and DKPs in foods are predominantly associated with bacteria involved in food spoilage or food-borne illness, whilst associations with AI-2-QSM appear more evenly split between instances of benign bacterial consortia and undesirable ones.

Different foods present distinct matrix effects, which will likely impact the stability and mobility of QSM produced by the microbiota within; high AI-2 activity is reported in frozen fish, tomato, cantaloupe, carrots, tofu and cow’s milk, whilst raw poultry, beef and artisanal cheeses appeared to inhibit AI-2 QS activity (Lu et al., 2004). This finding was supported by a more recent study measuring spiked AHL and AI-2 in water, skim milk and meat suspensions *via* a whole-cell biosensor assay, finding that signal was diminished only in the meat matrix (Wynn et al., 2018). The fluctuating levels of organic acids, pH and other biomolecules during food processing, especially in activities involving fermentation, are likely to influence QSM concentration and stability over time.

AI-2 appears to be particularly significant in the context of fermented food microbiomes. For instance, many bacteria isolated from the surface of smear-ripened cheeses produce AI-2, including *Arthrobacter nicotianae, Corynebacterium ammoniagenes, Corynebacterium casei, Microbacterium barkeri, Microbacterium gubbeenense and Staphylococcus equorum subsp. linens* (Novak and Fratamico, 2006; Gori et al., 2011). Another study of isolates from West African fermented foods reported AI-2 production in many Aerobic Endospore-producing Bacteria (AEB) such as *Bacillus subtilis, B. cereus, B. altitudinis, B. amyloliquefaciens, B. licheniformis, B. aryabhattai, B. safensis, Lysinibacillus macroides* and *Paenibacillus polymyxa* (Qian et al., 2015). This is supported by bioinformatic analysis of the microbiome of cocoa bean fermentations, which revealed a high prevalence of *luxS*-related genes in the core/soft-core of the lactic acid bacteria (LAB) *Limosilactobacillus fermentum, Pediococcus acidilactici* and *Lactiplantibacillus plantarum*. Additional *luxS* genes were also detected in some accessory genomes (de Almeida et al., 2021) and altogether participation in AI-2 signalling was proposed to help LAB adapt to diverse niches such as food fermentations.

AI-2 QS is also emerging as a key player in supporting desirable biofilms in food processing. For instance, AI-2 is present in high concentrations in Spanish table olive biofilms produced using a sequential yeast-LAB starter culture, which controlled undesirable spontaneous microbiota and maintained organoleptic quality throughout the product shelf life (Benítez-Cabello et al., 2020). In the same study, high AI-2 levels also correlated with high LAB counts on the olive skin surface, supporting the assertion that robust biofilm-forming capacity can enable potential probiotic strains to better survive and become established in fermented food microbiomes. This phenomenon has also been reported in other beneficial members of food microbiomes such as *Lactiplantibacillus pentosus* in olives (Perpetuini et al., 2016) and *Lactiplantibacillus paraplantarum* L-ZS9 in kimchi (Park et al., 2016). Thermal treatment is also highly relevant to food processing and whilst its effect on all QSM is not known, AI-2 concentrations are diminished by pasteurisation/ultra-heat-treatment (UHT; Lu et al., 2004).

An important limitation is that many studies report QSM production by strains isolated from foods, rather than quantifying QSM *in situ*, thereby only estimating the potential prevalence of QS in foods. A further confounding factor is the use of biosensors that may not distinguish between various enantiomers of QSM or between bacterial QSM and host-derived molecular mimics.

### Biofilms within food and food industries as environments for QS

Around one quarter of food waste is attributed to microbial processes (Huis in ‘t Veld, 1996), with implications for food safety and quality at a consumer level, economic losses at an industrial level and resource wastage in the context of sustainability. Microbial load and community composition are influenced by the nature and origin of raw materials, processing methods, transport and storage conditions (Jarvis et al., 2018; Van Reckem et al., 2020; Zwirzitz et al., 2020) and may fluctuate in accordance with the conditions of the niche. Food processing typically employs several physico-chemical hurdles to mitigate undesirable microbial growth such as thermal treatment, high pressure processing, use of acids, bacteriocins or other additives as well as regular sanitisation (Khan et al., 2017). Cumulatively, it has been proposed that this process could select for resistant strains that persist in the processing environment, particularly within biofilms, in turn re-contaminating finished foods (Walsh and Fanning, 2008).

Biofilms can be formed at many points in the food production process (Maes et al., 2019; Wagner et al., 2020), beginning with colonisation of raw plant materials and animal tissues during primary production. Biofilms can also form on man-made materials such as plastic or steel food-contact surfaces through preconditioning with biomolecules which facilitate attachment and extracellular matrix production by bacteria. Biofilm dwelling can provide a degree of shielding and protection from physical forces such as desiccation and shear, or biocides, facilitating greater resistance, particularly in mixed biofilms (Giaouris et al., 2015).

Biofilm formation has traditionally been viewed as a negative bacterial trait in the food industry, due to its association with specific spoilage organisms (SSO) and pathogenic bacteria that can re-contaminate foods post-processing, resulting in food spoilage and outbreaks of food-borne illness, as well as acceleration of ablation and corrosion of food-contact surfaces. The impact microbial transmission from biofilms into finished food products is of particular concern given the rising popularity of minimally processed and ready-to-eat foods (Meireles et al., 2018). Further, biofilms are increasingly recognised to promote antimicrobial tolerance (Uruén et al., 2020) as well as act as focal points for HGT between members of the population contained within (Abe, Nomura and Suzuki, 2020), potentially exacerbating the challenge of AMR.

However, biofilms also underpin and drive many key biotransformations in food and QS plays a role in at least some of these. Biofilm formation is thought to be crucial for the growth of milk kefir grains, which is catalysed by autoaggregation between LAB such as *L. lactis, Leuconostoc mesenteroides, Lentilactobacillus kefiri*, and *Lentilactobacillus sunkii* followed by synergistic kefiran production (EPS) by LAB and acetic acid bacteria (AAB), with cross-kingdom networking by yeasts such as *Kluyveromyces marxianus* (Wang et al., 2012; Han et al., 2018). Water kefir has received less attention than its dairy counterpart, but likely recapitulates this paradigm, with dextran-producing LAB thought to play a key role in grain growth (Fels et al., 2018; Guzel-Seydim et al., 2021). QS is likely to receive increased attention in this area, both for its relevance to the production of fermented foods with a biofilm quality such as kefirs, as well as its broader technological relevance in modulating the production of microbially synthesised extracellular polysaccharides.

Freeze-drying survival rates are also a concern in the commercial production of probiotic products. Notably, modulation of osmotic stress exposure in *L. plantarum* LIP-1 was found to enhance AI-2 production and promote biofilm formation by the strain, enabling it to better withstand the freeze-drying process (Jingjing et al., 2021). As subsequently outlined in the context of the human gut, biofilm formation and linked traits such as EPS production are increasingly being proposed as probiotic traits as they may enhance the survival of probiotic strains throughout gastrointestinal transit (Kelly et al., 2020).

### Bacteriocin production

Amongst the bacterial processes regulated by QS in foods, bacteriocin production has received particular attention due to its application in the production of food and feed, as well as antimicrobial therapy. Bacteriocins are a group of ribosomally synthesised peptides with antimicrobial activity, notably mediating interspecies competition due to immunity genes in the producer strain (Cotter et al., 2005). It has been approved as a natural biopreservative in foods by both the FDA in the US and EFSA in the EU and extensively investigated for applications in dairy foods, as well as meat, seafood and vegetable-based foods (Gharsallaoui et al., 2016).

As outlined previously, some AIP fulfil a dual role as both QSM and antimicrobial peptides, including lantibiotics (class I bacteriocins) such as nisin and lacticin 481, produced by some strains of *Lactococcus lactis* and subtilin, produced by specific strains of *B. subtilis* (Kleerebezem, 2004; Gobbetti et al., 2007). In the case of class II bacteriocins, such as certain plantaricins in *Lactiplantibacillus plantarum,* salivaricins in *Ligilactobacillus salivarius* and some carnobacteriocins produced by members of the genus *Carnobacterium*, a dedicated peptide pheromone is responsible for regulating transcription of the bacteriocin genes, forming a three-component system (Leisner et al., 2007; Maldonado-Barragán et al., 2009).

Bacteriocin production is by now well-established as an attractive feature in potential probiotic strains. In the context of interspecies interactions and microbial succession, bacteriocin production is proposed to enhance the viability of producer strains, particularly in complex ecosystems (Maldonado-Barragán et al., 2013). *In-situ* production of certain bacteriocins within foods can control the growth of spoilage and pathogenic microorganisms due to their specificity of action and non-toxic effect on the host (Dobson et al., 2012). Moreover, QS systems offer the potential to fine-tune the dynamics of bacteriocin synthesis. Future studies could examine in greater detail the dynamics of co-culture induction in complex communities, as reported in a strain-specific manner in *L. plantarum* (Maldonado-Barragán et al., 2013), with a view to optimising *in-situ* production of bacteriocins.

## Quorum sensing in the human gut microbiome

As previously described, the relatively high microbial load in the gut as well as the associations between the gut microbiome and health have made it a point of focus for human microbiome studies. The gut microbiome refers broadly to the gastrointestinal tract, primarily comprising the stomach, small and large intestine, sometimes also including the oral cavity and the microbial communities that become established within. The niches provided by the various compartments are highly dynamic with respect to changes in pH, oxygenation, nutrient availability, host–microbe interactions and microbial load, rendering community assemblage an incredibly complex ecological process (Donaldson et al., 2016). Additional spatial organisation is conferred by the distinct microenvironments provided by the gut lumen, mucin layer, intestinal crypts and mucosal epithelium.

Recent studies have highlighted the biofilm-like structure of mucus-associated bacterial communities in the gut lumen (Duncan et al., 2021), suggesting the likely prevalence of QS considering its previously established link with biofilms. Studies in this field are challenging due to the well-established inter-individual variation present even between ‘healthy’ individuals (Jones et al., 2018). Gaps remain in understanding the relative significance and importance of changes in composition and functionality of microbiomes as well as the ecological forces influencing assembly, maintenance and temporal fluctuation.

Perhaps the clearest instance of an interaction between microbiota present in a consumed food and the gut microbiome is food-borne illness. Many hurdles are mounted against invading bacterial pathogens, both from the resident microbiome (a phenomenon termed ‘colonisation resistance’) as well as host cells and immune mechanisms, all of which must be overcome to establish infection (Buffie and Pamer, 2013). In this delicate balance between colonisation, invasion and regulation, many bacterial pathogens utilise QS, at least in part, to regulate the expression of clinically relevant virulence factors that are metabolically costly and less strategic at low population densities (Rutherford and Bassler, 2012). Indeed, certain components of the human immune system are primed to detect and respond to pathogens and their virulence factors, which will be discussed below.

### Quorum sensing molecules detected in the human gut microbiome

**Figure 3:**
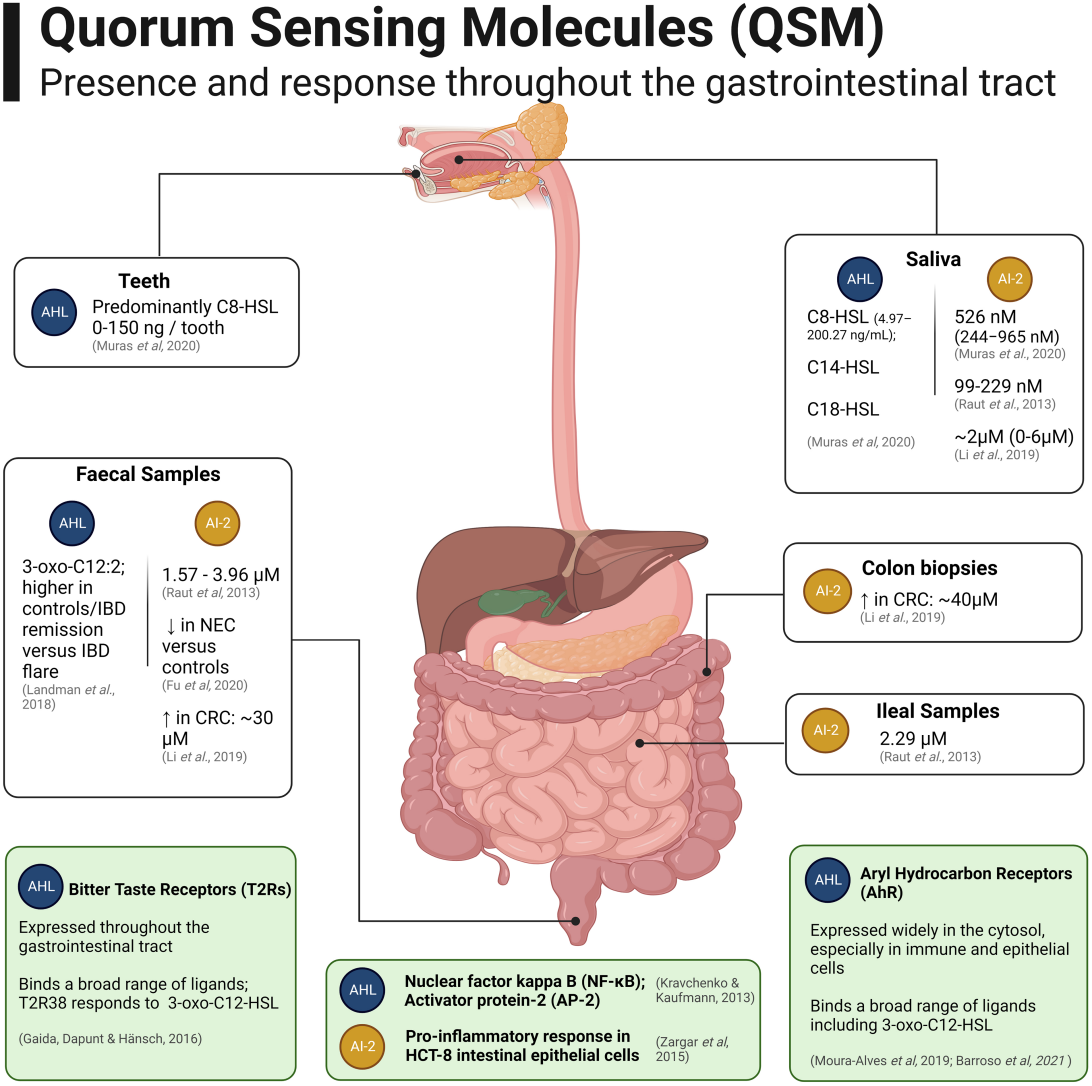
Quorum Sensing Molecules (QSM) which have been detected in samples from the human gastrointestinal tract (white boxes). Mammalian receptors present in the human gut capable of binding QSM as ligands are shown in light green boxes towards the bottom of the figure. Created with BioRender.com.

Relatively few studies have evaluated the presence of QSM within the human gut microbiome, due to the technical challenges associated with chromatography on luminal samples and low abundance of the molecules (Xue et al., 2021). Sampling of AHL-QSM at relevant temporal and spatial points is challenging due to their propensity to degrade at biological pH shortly after secretion, either spontaneously to tetramic acid, or by enzymatic hydrolysis of the lactone ring, leading their concentration to reflect temporal rather than historic population information (Peyrottes et al., 2020). Figure 3 provides an overview of the findings reported to date regarding QSM in the human gut.

With respect to the oral cavity, high-performance liquid chromatography-coupled mass spectrometry (HPLC-MS) has been used to detect AHL-QSM in extracted teeth and saliva samples, with C8-HSL being most abundant and present in all saliva samples and most of the extracted teeth, whilst C14-HSL and C18-HSL were present at lower levels (Muras et al., 2020).

Mass Spectrometry (MS) has been used to detect the novel QSM 3-oxo-C12:2, a derivative of the AHL 3-oxo-C12 bearing 2 unsaturations, in human faeces (Landman et al., 2018). It was detected at higher levels in both healthy control and Inflammatory Bowel Disease (IBD) patients in remission versus those in active flare and was correlated with higher abundances of Bacillota, especially *Faecalibacterium prausnitzii*, and lower abundances of *E. coli*. 3-oxo-C12:2 also displayed anti-inflammatory properties and an ability to limit cytokine-induced tight junction disruption in a Caco-2/TC7 model when compared with the native 3-oxo-C12 from *P. aeruginosa* (Aguanno et al., 2020). The presence and presumptive bioactivity in the human gut microbiome of AHLs bearing such unsaturations is intriguing, as unsaturated pheromones are generally uncommon and their production had otherwise only been reported in the marine *Roseobacter* clade (Ziesche et al., 2015; Peyrottes et al., 2020). The producer of the precursor AHL and unsaturations, as well as its precise biological effects, remain unidentified to this date. A novel UPLC-MS/MS method has recently been developed to detect AHL-QSM in caecal, sera and liver samples of germ-free, conventional and *Citrobacter rodentium*-infected mice (Xue et al., 2021). This study demonstrated the translocation of bacterial AHL-QSM across the intestinal barrier throughout host tissues and the perturbation in AHL profiles provoked by *C. rodentium* infection.

Relatively few studies have investigated AI-2 levels in the gut microbiome. Liquid chromatography–tandem mass spectrometry was used to detect AI-2 produced by the oral microbiota in saliva samples (Campagna et al., 2009) *via* the linear precursor molecule 4,5-dihydroxy-2,3-pentanedione (DPD). Concentrations fluctuated considerably between participants (average 526 nM, range 244–965 nM), potentially highlighting inter-individual variation or temporal fluctuations in the oral microbiome (Rickard et al., 2008). AI-2 also appears to mediate inter-kingdom interactions in the human mouth in that, for example, hyphal development in the fungal pathogen *Candida albicans* is inhibited by AI-2 produced by *Aggregatibacter actinomycetemcomitans* (Bachtiar et al., 2014). Beyond the oral cavity, AI-2 has been detected in ileal and rectal samples taken during endoscopy from participants with and without IBD (Raut et al., 2013). In the gut, one study found diminished AI-2 levels in the acute phase of Necrotising Enterocolitis (NEC), a serious gastrointestinal disease primarily affecting infants, consequentially proposing the QSM as a potential biomarker for early detection and prophylaxis (Fu et al., 2020). In contrast, AI-2 levels were observed to be significantly higher in faecal samples and biopsies from patients with Colorectal Cancer (CRC) versus those of adenomas/healthy controls (Li et al., 2019a,b).

AI-3 is the most recently described QSM thought to be found in the gut. AI-3 family pyrazinones are produced by a wide range of bacteria relevant to the gut microbiome, as well as in pathogen-free mice colonised with *E. coli* BW25113, although human microbiome samples have not been interrogated to date using analytical techniques (Kim et al., 2020). Similarly, although direct quantification of other QS systems has not to our knowledge been reported, *in silico* predictions *via* the QSHGM database (Wu et al., 2021b) reveal presence of photopyrone, dialkylresorcinol and DSF genetic circuits in a wide range of gut microbes, although this remains to be evaluated using analytical techniques.

Taken together, QS systems appear to be active at various points of the human gut microbiome as are several distinct strategies by the eukaryotic host to monitor and respond to bacterial QSM. Future studies leveraging novel sampling approaches can further clarify the relative importance and clinical significance of the various systems, particularly regarding the interactions between commensals and opportunistic pathogens introduced to the gut microbiome.

### Qs influence on collective bacterial behaviours in the gut

Lifestyle switching, through environmental and quorum sensing, has been proposed to favour the survival and colonisation of introduced species in the human gut (Mukherjee and Bassler, 2019). QS can also facilitate the establishment of collective behaviours of microbial populations and communities within the gastrointestinal tract, potentially affecting community assemblage and host physiology. AI-2 systems are most commonly implicated in this context, in both positive and negative senses.

*Vibrio cholerae* provides one of the clearest examples of lifestyle switching in order to adapt to and survive harsh environmental conditions in the environment and in the gut using QS. It forms aggregates of biofilms when surrounded by other *V. cholerae* cells and switches to a dispersal strategy to escape the matrix when surrounded by high numbers of ‘other’ non-*V. cholerae* cells. (March and Bentley, 2004; Bari et al., 2013; Bridges and Bassler, 2021). It has recently been proposed that *V. cholerae* senses its own quorum *via* CAI-1 QSM in conjunction with the biogenic amine norspermidine, which is relatively specific to *V. cholera*, whilst other populations are monitored using AI-2 and spermidine, a ubiquitous biogenic amine found throughout the human gut (Bridges and Bassler, 2021). CAI-1 has also been demonstrated to enhance the virulence of the non-producer Enteropathogenic *E. coli* (EPEC), possibly contributing to co-infection of the host (Gorelik et al., 2019). AI-2 also modulates sporulation and biofilm formation in the pathogenic *Clostridium difficile,* facilitating switching between dormancy, vegetative growth, virulence, immune evasion and antibiotic tolerance (Ðapa et al., 2013; Desai and Kenney, 2019; Tijerina-Rodríguez et al., 2019). Additionally, *C. difficile* uses an *agr* system mediated by thiolactone QSM to regulate production of the toxins TcdA and TcdB which compromise the host epithelial barrier in *C. difficile* infection (CDI; Darkoh et al., 2015). Certain *C. difficile* phage appear to bear and disseminate *agr* variants amongst their hosts, highlighting the complexity of microbiome dynamics and the difficulty in limiting investigations to single kingdoms (Hargreaves et al., 2014). This also supports the recent implication of bacterial QS as a broad mediator of phage-host interactions in the gut (León-Félix and Villicaña, 2021). Such insights certainly require additional scrutiny, but could prove significant in the development of phage therapy.

There are also many instances where QS activity is associated with positive compositions or outputs from microbial communities. One standout study found that increasing local levels of AI-2 in a mouse model of antibiotic treatment promoted the recovery of Bacillota (previously known as Firmicutes) whilst hindering the expansion of Bacteroidota (previously known as Bacteroidetes; Thompson et al., 2015). As previously described for food-borne probiotic strains, AI-2 QS was also shown to positively influence colonisation of a probiotic *Bifidobacterium breve* UCC2003 in the gut (Christiaen et al., 2014). Indeed, *luxS-*deficient *Streptococcus mutans* mutants incapable of producing AI-2 were found to upregulate carbohydrate metabolism and ABC transporters, highlighting the potential for QS to govern individual behaviours as well as collective ones (Yuan et al., 2021) and underlining the necessity for systems biology approaches when evaluating proposed therapeutic applications of QSI.

### Detection of bacterial QS by the human host

Bacterial quorum sensing may also contribute to host–microbe interactions as evidenced by the existence of pathways in eukaryotic multicellular organisms to detect and even interfere with microbial communications (Aframian and Eldar, 2020). Indeed, the broader field of microbial endocrinology abounds with examples of crosstalk between host hormones and bacterial signalling systems, perhaps unsurprisingly considering the hormone-like properties of bacterial QSM (Neuman et al., 2015; Li et al., 2019a). Taken together, this is suggestive of co-evolution and adaptation by the eukaryotic host to monitor for AHL production and coordinate a defensive response.

AHL-QSM are small, lipophilic molecules and although poorly soluble in water, their diffusion into eukaryotic cells has recently been demonstrated, with interaction with intracellular targets also considered possible (Kamaraju et al., 2011; Peyrottes et al., 2020). Molecular self-assembly in aqueous solution into micelles and vesicles has been observed for 3-oxo-C8-AHL, 3-oxo-C12-AHL, C12-AHL and C16-AHL, which may guide our understanding of the kinetics of secreted QSM (Gahan et al., 2020). Bacterial outer membrane vesicles (OMVs) have also been proposed to traffic quorum sensing molecules within and between bacterial microcolonies, circumventing the issue of hydrophobicity (Toyofuku et al., 2017). OMVs have also been detected in host blood, heart and urine samples, being able to cross the mucus layer and epithelium of the gut (Stentz et al., 2015). Whilst OMVs have been studied to the greatest extent in the context of Gram-negative pathogens, attention is turning to their significance in probiotic and commensal strains, including *E. coli* Nissle 1917 and ECOR63, respectively (Alvarez et al., 2016), however more precise characterisation of their composition and relevance to QS is required.

Bitter taste receptors (T2Rs) belong to the G protein-coupled receptor group and were initially studied in the context of taste, being highly expressed on the tongue. However they have been found to be expressed in a variety of other sites including the upper respiratory tract and gastrointestinal tract, suggesting a broader role (Verbeurgt et al., 2017). Indeed, there is evidence of variable interaction of these receptors with numerous bacterial metabolites including AHLs. T2R38 is one example that is expressed throughout the gastrointestinal tract on intestinal epithelial cells (Behrens and Meyerhof, 2011; Karlsson et al., 2012) and activated by the AHL-QSM 3-oxo-C12-HSL in myeloid cells, enhancing phagocytic activities (Gaida et al., 2016) − although the myeloid cells in the latter study were derived from patient biopsies of osteomyelitis. Yet another study, using a heterologous expression system, found that T2R38 did not respond to 3-oxo-C12 HSL nor the cyclic AIP Agr-D1 thiolactone and CSP-1, but was activated by a range of bacterial metabolites including acetone, 2-butanone, 2-pentanone, 2-methylpropanal, dimethyl disulphide, methylmercaptan, and γ-butyrolactone (Verbeurgt et al., 2017). Methodological differences between the studies may account for this discordance.

Pro-inflammatory cascades have also been reported by AHL interaction with nuclear factor kappa B (NF-κB) and activator protein-2 (AP-2) receptors (Kravchenko and Kaufmann, 2013). The human aryl hydrocarbon receptor (AhR) is present in the human gut and interacts with a wide array of microbial metabolites and was shown to detect and react to relative abundances of AHLs, quinolones and phenazines produced by *P. aeruginosa* to manage immune response over the course of infection (Moura-Alves et al., 2019; Barroso et al., 2021).

In contrast to AHL-QSM, unmodified AI-2 is hydrophilic and does not appear to penetrate the plasma membrane, although heptyl modifications increase its affinity (Kamaraju et al., 2011). *In vitro* exposure of HCT-8 intestinal epithelial cells to AI-2 was found to provoke an inflammatory response (Zargar et al., 2015); this interaction could be studied in a more representative model system such as intestinal organoids or *in vivo*. Recent evidence suggests that mammalian epithelial cells can produce an AI-2 analogue that may mimic and interfere with bacterial QS, although the structure has yet to be determined (Ismail et al., 2016).

## Quorum sensing inhibition in food microbiomes

Given the association between QS and bacterial biofilm formation and food spoilage activity, QSI has been proposed as a potential strategy to positively regulate bacterial phenotypes and improve food safety and quality. In fact, many foods themselves exhibit QSI activity associated with defence systems in plant/animal tissue or with the presence of microorganisms that have developed mechanisms to interfere with QS activity of neighbouring species.

Quorum sensing systems are interdependent and complex—the field of QSI can generally benefit from the application of a systems biology approach to understand the network of interactions occurring within a particular microbiome. As applied to food production systems, QSI interventions may need to be timed to suppress bacterial QS at critical control points in the food chain, as opposed to their constitutive deployment, or targeted against specific bacterial species implicated in spoilage or pathogenesis whilst maintaining the structure of the overall food microbiome.

### QSI produced in microbe-microbe interactions in food microbiomes

**Figure 4:**
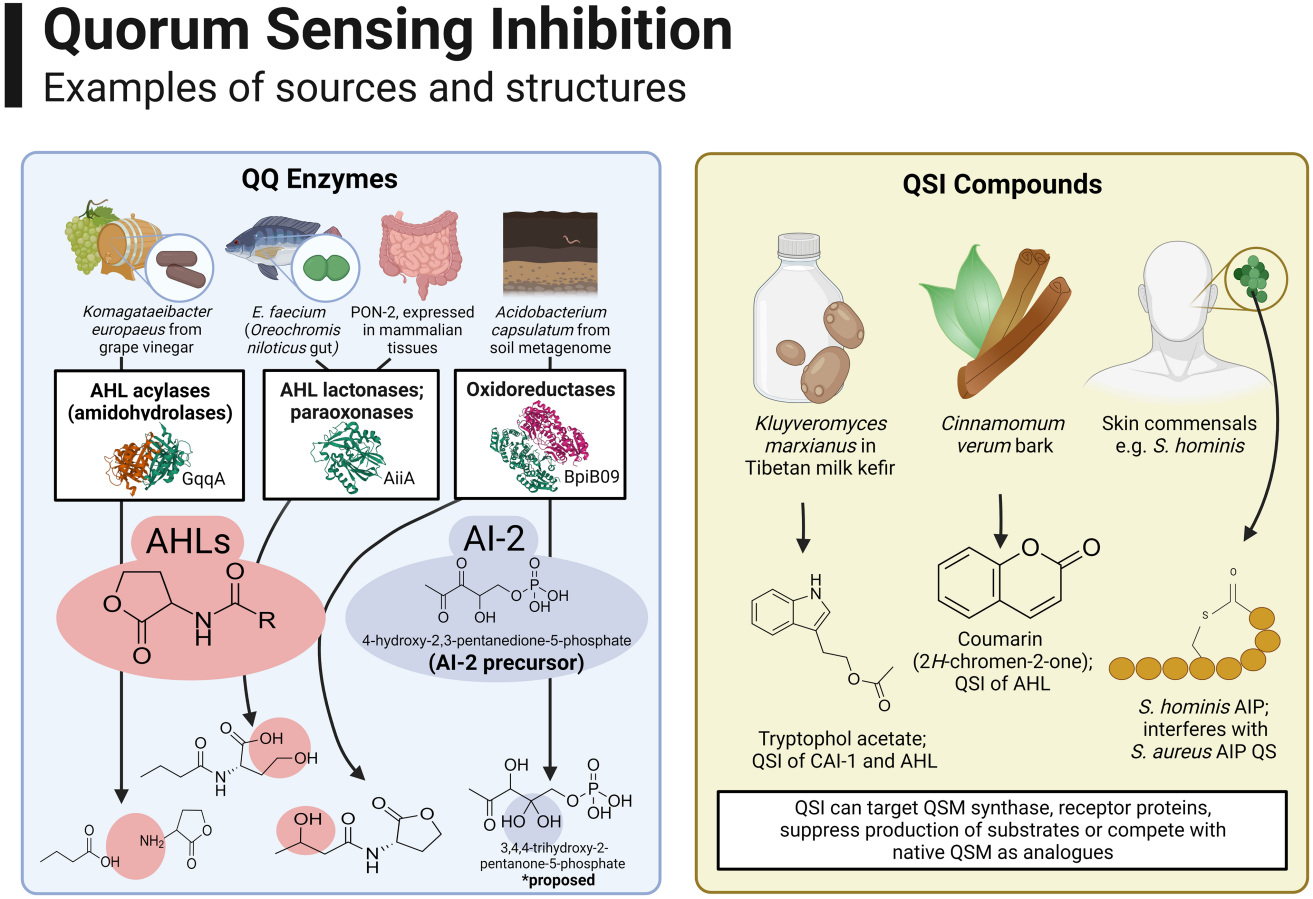
Overview of the source and structures of some prominent examples of QQ enzymes and QSI compounds. Many QQ enzymes have been described for AHLs, such as the AHL acylase GqqA reported in *Komagataeibacter europaeus* isolated from grape vinegar [Werner et al., 2021; Image from the RCSB PDB (rcsb.org) of PDB ID 7ALZ], AHL lactonases such as AiiA from *E. faecium* isolated from the gut of *Oreochromis niloticus* [Vadassery and Pillai, 2020; Image from the RCSB PDB (rcsb.org) of PDB ID 7L5F] and paraoxonase enzymes which are expressed widely in mammalian tissues (Peyrottes et al., 2020). Oxidoreductase enzymes have been isolated from environments such as soil and activity has been reported against AHLs (Bijtenhoorn et al., 2011; Image from the RCSB PDB (rcsb.org) of PDB ID 3RKR) as well as AI-2 (Weiland-Bräuer et al., 2016). Examples of non-enzymatic QSI include tryptophol acetate, active against CAI-1 and AHLs (Malka et al., 2021), coumarin and derivatives, which can interfere with AHL-QS (Gutiérrez-Barranquero et al., 2015) and AIP analogues produced by closely related staphylococcal species (Peng et al., 2019). Created with BioRender.com.

Several instances of QSI activity in foods are attributed to members of food microbiomes. Many quorum quenching (QQ) enzymes belonging to AHL lactonase, acylase and oxidoreductase families have been identified, for the most part being isolated in soil and marine metagenomes, plant symbionts and pathogenic bacteria (Bijtenhoorn et al., 2011; Fetzner, 2015). Figure 4 provides an overview of the source and structures of some prominent examples of QQ enzymes and QSI compounds. The majority of QSI discovered to date are active against AHL-QSM; to our knowledge, only one AI-2 QQ enzyme has been identified to date, originating in a soil metagenomic bank and hypothesised to reduce an AI-2 precursor, 4-hydroxy-2,3-pentanedione-5-phosphate, to the inactive derivative 3,4,4-trihydroxy-2-pentanone-5-phosphate (Weiland-Bräuer et al., 2016). For an extensive list of microbially derived QQ enzymes, the reader is directed to a comprehensive review (Sikdar and Elias, 2020).

With that being said, food microbiomes have yielded some novel QSI agents in recent years, particularly QQ enzymes as shown in Figure 3. An AHL acylase with was recently characterised in a *Komagataeibacter europaeus* strain isolated from grape vinegar (Werner et al., 2021), complementing an earlier report of another putative AHL acylase in a *S. epidermidis* strain isolated from fermented soybean curd (Mukherji and Prabhune, 2015). In the latter, the authors propose QQ as a possible probiotic trait in food fermentations to reduce overall concentrations of AHL-QSM. Another study demonstrated the potential of *in silico* screening through mining of a Mao-tofu metagenome, discovering a novel AHL lactonase (aii810) active at low temperatures (Fan et al., 2017).

As AHL-QS producers account for a large proportion of the burden of spoilage and pathogenesis in aquaculture, this area has received significant attention and yielded an array of novel AHL-QQ enzymes. Aquaculture and fish gut microbiome isolates including *Bacillus* sp. QSI-1, *Bacillus licheniformis* T-1 and *Enterococcus faecium* QQ12 have been identified as potential probiotic feed ingredients due to their potential modulatory role for the AHL-QSM profile of the fish gut microbiome (Chu et al., 2014) and protecting against *A. hydrophila* infection through AHL lactonase activity of YtnP (Peng et al., 2021) and autoinducer inactivation (*aiiA*) homologues (Vadassery and Pillai, 2020). Another study found widespread AHL-QQ activity in bacterial isolates from rainbow trout tissue, including members of the genera *Bacillus*, *Enterobacter*, *Citrobacter*, *Acinetobacter*, *Agrobacterium*, *Pseudomonas* and *Stenotrophomonas*. Characterisation showed variability in both enzymatic and non-enzymatic modes of action, highlighting the diversity of QQ activity observed in the comparably simple fish gut microbiome, as well as a propensity for cell-bound activity rather than secretion (Torabi Delshad et al., 2018).

Metagenomic studies have revealed the presence of potential quorum quenching determinants in dairy products and production plants (Alexa Oniciuc et al., 2020); food microbiomes may represent in this sense a relatively untapped reservoir for novel QSI determinants. Indeed, there are numerous reports of culture extracts from specific LAB strains exerting anti-biofilm, anti-virulence and QSI effects on other bacterial species; for instance, cell free supernatants of LAB isolated from fermented grape interfere with AI-2 quorum sensing and biofilm formation in *Salmonella* Typhi and *S.* Typhimurium (Pelyuntha et al., 2019) and supernatants of LAB strains isolated from beef and cow’s milk inhibited pyocyanin production in a *P. aeruginosa* isolate of rancid butter and diminished violacein production in *C. violaceum* (Aman et al., 2021). The molecular mechanism of this widespread yet strain-specific activity remains unelucidated to date, but is thought to involve, at least in part, acid production along with small molecules secreted by the LAB strains. Indeed, another study evaluated both acidic and neutralised supernatants of LAB strains, finding that neutralised supernatants more weakly inhibited biofilm formation in *P. aeruginosa* compared with acidic fractions; moreover, only acidic supernatants interfered with the *las* and *rhl* quorum sensing systems (Rana et al., 2020).

Intriguingly, many examples of QSI arise amongst eukaryotic members of food microbiomes, specifically fungi. AI-2 QSI activity of brown algae has been attributed not to the plant cells but rather to bacterial and fungal endophytes (Tourneroche et al., 2019). Recently, a novel QSI compound (tryptophol acetate) was discovered to be produced by the yeast *Kluyveromyces marxianus* present in a Tibetan milk kefir (Malka et al., 2021). It was found to interfere with bacterial AHL and CAI-1 signalling, with possible applications for bioprotection of foods and prevention of proliferation of enteric pathogens such as *V. cholerae* (CAI-1). Again, this highlights the need for systems biology approaches that encompass all relevant kingdoms of life, not solely bacteria, when studying QS in a microbiome context.

Lastly, purified microbially derived enzymes can be incorporated into food formulations or cleaning protocols for food processing plants. To our knowledge, just one study has examined the use of enzymes with quorum quenching activity in foods. In this case, it was found that treatment with a commercial preparation of 7 peptidases and 1 amylase (Flavourzyme) decreased the relative expression of genes involved in quorum sensing (Nahar et al., 2021). However, this study only examined AI-2 related transcription, which could be due to suppression of the AMC (Activated Methyl Cycle) as a result of global metabolic perturbation. Treating raw sturgeon with AiiAAI96 (AHL lactonase) and nisin (bacteriocin) prior to vacuum packaging extended the product shelf life by 5 days (Gui et al., 2021), showing promise as a biopreservative targeting both Gram-positive and negative SSO. Heterologous expression of QQ enzymes *in situ* offers another potential route for biotechnological applications, as exemplified by the use of a *Lacticaseibacillus casei* strain, often used in the dairy industry, to express the AHL lactonase AiiK which can attenuate virulence in *Aeromonas hydrophila* (Dong et al., 2020) and *P. aeruginosa* (Dong et al., 2018).

### QSI By components of food matrices

In addition to microbially produced QSI molecules, it has been established that many components of food matrices can, to some degree, modulate bacterial QS. A brief overview of prominent examples are provided in Table 2. In some cases, such as the organic acids, they may affect the stability of the QSM themselves or downregulate their synthesis by the producer strain due to acid stress. In other instances, such as fatty acids, the QSI phenomenon is thought to arise due to their structural similarity to the fatty-acid-derived QSM including AHLs and DSF. Indeed, in the case of ascorbic acid (vitamin C) the mechanism of action could arise due to its effect on pH or due to the structural similarity between it (*5S*)-5-[(*1R*)-1,2-Dihydroxyethyl]-3,4-dihydroxy-2(*5H*)-furanone) and other furanones – such disambiguation will be critical for harnessing it as a potential QSI agent.

**Table 2:** Overview of components of food matrices which can mediate bacterial Quorum Sensing Inhibition (QSI) or related phenotypes, such as biofilm inhibition and sporulation.

Food component	Examples	QS system or phenotype affected	Bacteria affected	Concentration	References
Organic acids	Lactic acid	AHL; Anti-biofilm	*Chromobacterium violaceum* CV026; *E. coli*; *Salmonella* sp.	0.2–1%	Amrutha, Sundar and Shetty, (2017)
	Acetic acid	AHL; Anti-biofilm	*C. violaceum* CV026; *E. coli*; *Salmonella* sp.	1–1.5%	
	Citric acid	AHL; Anti-biofilm	*C. violaceum CV026, E. coli*; *Salmonella* sp.	1.5–2%	
	Malic acid	AI-2	*E. coli O157:H7; S.* Typhimurium	4%	Almasoud et al., (2016)
	Ascorbic acid (vitamin C)	AHL	*P. aeruginosa*	5–12·5 mg/ml	El-Mowafy, Shaaban and Abd El Galil, (2014); Pandit et al., 2017)
		Sporulation; AI-2	*C. perfringens,*	10 to 30 mM (sporulation); 300 mM (AI-2)	Novak and Fratamico, (2006)
		AI-2	*E. coli* EMC17	125 mM	Shivaprasad et al., (2021)
		AIP (ComC)	*B. subtilis*	10 to 40 mM	Pandit et al., (2017)
Long Chain Fatty Acids (LCFAs)	Mono-unsaturated palmitoleic myristoleic acids	AHL	*Acinetobacter baumannii*	0.02 mg/ml	Nicol et al., (2018)
	Linoleic acid	DSF	*P. aeruginosa*	10 μM	Kim et al., (2021)
	Fatty acids produced by fungal endophyte of *Coriandrum sativum*	Anti-biofilm	*S. mutans*	31.3 mg/l	Abdel-Aziz, Emam and Raafat, (2020)
	Oleic acid (Cis-9-octadecenoic acid)	Anti-biofilm	*S. aureus*	0.1% v/v	Stenz et al., (2008)
		AHL	*C. violaceum* CV026;	3.696 mg/ml	Singh et al., (2013)
	Palmitic acid (C16:0), Stearic acid (C18:0), Oleic acid (C18:1ω9), Linoleic acid (C18:2ω6)	AI-2	*V. harveyi, E. coli*	1–10 mM	Soni et al., (2008)
Endocannabinoids	Endocannabinoid 2-AG (arachidonic acid derivative)	AI-3 (*via* QseC)	EHEC, *Citrobacter rodentium*	Not quantified – 2-AG levels were elevated in intervention group *via* knockout of monoacylglycerol lipase (Mgll)	Ellermann et al., (2021)
Furanones	Brominated, halogenated and natural forms in seaweeds, tomatoes, strawberries and other berries; synthetic derivatives	AHL	*P. aeruginosa*	3.125–50 μM	Colin Slaughter, (1999); Manefield et al., (1999); Proctor, McCarron and Ternan, (2020)
		AI-2	*Vibrio harveyi, Vibrio parahaemolyticus*	20 mg/l	Defoirdt et al., (2006)
		Anti-biofilm	*S.* Typhimurium; *L. monocytogenes;*	10 to 15 μM (*S.* Typhimurium); 0.05 mmol/l at 24 h, 2 mmol/l at 48 h (*L. monocytogenes*)	Janssens et al., (2008); Rodríguez-López et al., (2019)
Coumarin	Dihydrocoumarin	Anti-biofilm	*Hafnia alvei*	3.2 mM	Hou et al., (2017)
		AHL	*C. violaceum* CV026	6.3 mM	
	Coumarin from *Cinnamomum verum*	AHL	*P. aeruginosa* (*pqsA, rhlI, lasI*)	1.36 mM	Gutiérrez-Barranquero et al., (2015)
Vitamin B2	Riboflavin and derivatives	AHL (LasR system)	*P. aeruginosa*	200 μM (effective soluble concentration)	Rajamani et al., (2008)
Steviol glycosides	Steviol	AHL (*las / rhl* systems)	*E. coli* K802NR-pSB1075 (*las*); *P. aeruginosa* PAO-JP2 (pKD-rhlA) (*rhl*)	0.26–0.52 mM (*las*); 0.0325–0.52 mM (*rhl*)	Markus et al., (2020)
	Reb A			0.019375–0.31 mM (*rhl*)	
	Stevioside			0.5–1.0 mM (*las*); 1 mM (*rhl*)	

Generally, plant-based foods do not appear to support bacterial quorum sensing activity to the same extent as animal-based ones (Medina-Martínez et al., 2006). This phenomenon is hypothesised to arise from co-evolutionary forces between plants and microorganisms and is primarily mediated by inhibitory plant secondary metabolites including furanones, phenolics and other bioactives that can act as QSM analogues (Rodrigues et al., 2016; Zaytseva et al., 2019; Pun et al., 2021). This could suggest the potential for plant-derived ingredients to generally disrupt bacterial QS activity or support their incorporation in food formulations to modulate the food microbiome of the product. However due to the general bioactivity of plant secondary metabolites, possible off-target effects should be assessed and effective QSI activity established in the context of the matrix effects and digestion.

Whilst food matrix-derived peptides may be similar in size and sequence to known QS AIP, no QSI peptides have been described to date in foods. Bioactive peptides are intriguing due to their small size, which allows them to diffuse relatively easily through food matrices and biofilms; additionally, they are amenable to bioengineering against resistance as they are gene-encoded (Bhutia and Maiti, 2008; Field et al., 2019). QSI AIP have been described in coagulase-negative staphylococci in the skin microbiome with activity against *S. aureus* (Peng et al., 2019) − this could suggest that intra-genus competition within food microbiomes represents an opportunity for future mining and discovery of potential therapeutic agents with direct applications in food production.

This provides a new perspective on food processing and formulation and the impact of ingested foods on the gut microbiome. Whilst many components pertain to raw food ingredients derived from plants and animals, many of these such as organic acids, fatty acids and vitamins can themselves be microbially synthesised and are of interest in their own right in the context of human nutrition. Aromatic and volatile compounds are overrepresented, with many QSI compounds also having a role in flavouring such as Maillard reaction-derived furanones (Wang and Ho, 2008) and lactone derivatives such as coumarins. Understanding the directionality of these effects, particularly with respect to stability and efficacy in food matrices and withstanding transit through the digestive tract, will be of key importance in the development of applications in the food formulation space.

## Quorum sensing inhibition in the human gut microbiome

As previously outlined, quorum sensing has been implicated in the onset of pathogenesis in infections and is thought to influence community composition in other human-associated microbiomes such as those of the oral cavity, lung, skin, urinary and reproductive tracts (Bradshaw and Sobel, 2016; Cole et al., 2018; Scoffone et al., 2019; Williams et al., 2019). As some pathogenic bacteria utilise QS to time their deployment of virulence factors, it has been proposed that QS activities of neighbouring bacteria in the gut microbiome could interfere with this timing, either by QSI or by prematurely inducing the quorum level with species-agnostic QSM (Cho et al., 2021). Unsurprisingly, many instances of QSI activity can be observed in the crosstalk between host and microbe and in communications within bacterial communities.

### QSI enzymes in host tissue

Paraoxonase (PON) enzymes are expressed widely across human tissues, including the intestines and can inactivate AHL-QSM through hydrolysis of the lactone ring moiety (Camps et al., 2011; Peyrottes et al., 2020). Like many QQ enzymes, PON have a broad substrate specificity and can also inactivate esters, e.g. oestrogen esters, lactones or organophosphates, highlighting the need to consider off-target effects in evaluation of QS-based therapies.

### QSI produced in microbe-microbe interactions in the human gut

Microbe-microbe interactions in the human gut can involve cooperation, through sharing of resources and cross-feeding, as well as antagonism, in efforts to outcompete neighbouring cells. Just as in food microbiomes, QS appears to be widespread amongst bacteria in human gut microbiomes and likely, to some degree, mediates interspecies interactions therein. Indeed, AI-2 was shown to orchestrate both cross-feeding and also QSI in co-culture of *C. acetobutylicum* and *D. vulgaris* Hildenborough, depending on the extent of nutrient limitation in the system (Ranava et al., 2021), highlighting the conditionality which can be present in bacterial cooperation.

Overall, there are relatively fewer instances of QQ enzymes or QSI compounds being reported from the gut microbiome, possibly due to the focus of bioprospecting efforts on natural environments, as well as the difficulty in successfully culturing gut commensals *in vitro*. However, QSI potential has been established by some studies, for example, in the oral microbiome 60% of recovered bacteria in dental plaque and saliva exhibited AHL-degradation capacity and treatment of mock communities with the broad-spectrum AHL-lactonase Aii20J reduced biofilm formation and altered the overall community composition (Muras et al., 2020). Penicillin V acylases are microbially produced enzymes within the choloylglycine hydrolase family able to inactivate AHL-QSM due to structural similarity with the functional residues of native AHL acylases (Liu et al., 2012; Mukherji and Prabhune, 2015; Daly et al., 2021). To date, however, there are scarce reports of their presence in human gut microbiomes wherein bile salt hydrolases predominate. This highlights the importance of substrate specificity and receptor promiscuity when potentially targeting QS in complex microbiomes such as those of foods and the gut.

In addition to enzymatic QQ, there are several reports of QSM produced by one species acting as analogues for other species, potentially mediating microbe-microbe interactions. Whilst, to our knowledge, no direct examples exist pertaining to the human gut microbiome, there are examples from the human skin and anaerobic waste digestion microbiomes. AIP produced by skin commensals such as coagulase-negative staphylococci including *Staphylococcus hominis* can inhibit the *agr* system of *S. aureus,* reducing the expression of phenol-soluble modulin α (PSMα) which undermines skin barrier integrity (Williams et al., 2019).

Whilst, to our knowledge, no clinical trials have been conducted to date in humans, engineered probiotics are being evaluated to harness QS to promote human health and wellbeing. An *E. coli* Nissle 1917 strain designed to respond to the AHL-QSM 3OC_12_HSL was shown to eliminate and prevent *P. aeruginosa* gastrointestinal infections in animal models (Hwang et al., 2017). In another study using a rational design approach, expression of small peptides resembling LuxS active sites reduced AI-2 activity of aquaculture pathogens *Edwardsiella tarda*, *A. hydrophila* and *V. harveyi* (Sun and Zhang, 2016). Given the ubiquity of AI-2 signalling, this approach clearly offers broad cross-disciplinary applicability.

Another approach involves direct oral administration of purified QQ enzymes such as AHL-lactonase, which resisted digestion and attenuated virulence of *A. hydrophila* in a zebrafish model (Cao et al., 2012). Studies examining feasibility of QSI therapies as applied to human gut microbiomes must take digestive processes into account to ensure delivery to the site of action, as well as evaluating the impact on the structure and composition microbiome. *Ex vivo* modelling of the human gut offers great promise in modelling the effect of QQ enzyme treatment on the functional and taxonomical composition of the gut microbiome.

## Conclusion

The field of quorum sensing research has seen a shift from fundamental to translational science in the past two decades. Nevertheless, despite a preponderance of newly discovered and/or bioengineered quorum sensing inhibitors, several important gaps remain to be addressed so that the therapeutic targeting of quorum sensing systems for the modulation of food and human gut microbiomes may expand as a field.

Firstly, analytical techniques must continue to be developed to augment our capacity to monitor quorum sensing activity and efficacy of quorum sensing inhibitory interventions, particularly for AIP-QS systems and QS within complex matrices such as foods and luminal samples from the human gut. Continued innovation with respect to bioinformatic approaches in particular can further potentiate our understanding of the significance of QS within microbiomes generally, not restricted to those discussed in this review. In this sense, an important consideration in future study design will be the disambiguation of QS gene presence/absence and QS functionality in food and gut microbiomes. Whilst biofilms pose challenges with respect to modelling in a meaningful manner, their ubiquity and potential to act as reservoirs for AMR genetic determinants, food-borne pathogens and specific spoilage organisms are likely to drive efforts to better understand, manage and harness biofilms in foods and in the human gut.

Secondly, many interventions such as specific strains, probiotics, prebiotics and phage, are being investigated with a view to the modulation of complex microbiomes such as those of foods and the human gut. As described earlier, quorum sensing-regulated biofilm production has been shown to improve the survival of probiotic strains during food processing, food fermentations and transit through the human gut. We propose that future studies in this field may consider quorum sensing activity in specific strains as a probiotic trait, where supported by evidence and subject to requisite evaluation. Similarly, strains with QS inhibitory activity could be investigated for their potential clinical or biotechnological applications.

Thirdly, our ability to address AMR can be augmented by better understanding the role of QS in disseminating AMR genes, as well as evaluating QSI agents with a view to the development of anti-virulence therapies and combinatorial agents to enhance the efficacy of existing antibiotics. Here, harnessing systems biology and synthetic ecology approaches will be critical to ensure only the most effective and promising hits are brought through to the preclinical stage.

In conclusion, over the span of a little over 50 years, the field of quorum sensing research has been founded and come to be regarded to be of key importance with respect to microbial physiology in the context of social interactions. Whilst food and human gut microbiomes have received relatively less attention, the accumulated literature points to the presence of quorum sensing molecules and regulated behaviours in these contexts. Further, the abundance and diversity of host receptors to survey bacterial QSM, in addition to the numerous examples of QSI systems across kingdoms of life and environmental niches, has likely arisen due to the ubiquity and evolutionary importance of QS within microbiome science. Addressing the knowledge gaps identified above will help in the critical evaluation of proposed QS and QSI interventions in these contexts, with the potential to positively impact human, animal and ecosystem health.

## Author contributions

AF, AA-O, CG, and PC conceived the manuscript. KF wrote the manuscript. AA-O, CG, AF, and PC contributed to the final preparation. All authors contributed to the article and approved the submitted version.

## Funding

This research was conducted with the financial support of Science Foundation Ireland (SFI) under Grant Number SFI/12/RC/2273 P2.

## Conflict of interest

The authors declare that the research was conducted in the absence of any commercial or financial relationships that could be construed as a potential conflict of interest.

## Publisher’s note

All claims expressed in this article are solely those of the authors and do not necessarily represent those of their affiliated organizations, or those of the publisher, the editors and the reviewers. Any product that may be evaluated in this article, or claim that may be made by its manufacturer, is not guaranteed or endorsed by the publisher.

## Figures and Tables

**Table:** 

agr	Accessory gene regulator system
AHL	Acylated homoserine lactone
AhR	Aryl hydrocarbon receptor
AI	Autoinducer
AIP	Autoinducer peptide
AMC	Activated methyl cycle
AMR	Antimicrobial resistance
ATP	Adenosine triphosphate
CAI-1	Cholera autoinducer
cAMP	Cyclic adenosine monophosphate
c-di-GMP	Cyclic diguanylate (c-di-GMP)
CRC	Colorectal cancer
DKP	Diketopiperazines
DPD	4,5-dihydroxy-2,3-pentanedione
DPO	3,5-dimethylpyrazin-2-one
DSF	Diffusible signal factor
EHEC	Enterohemorrhagic *Escherichia coli*
EPEC	Enteropathogenic *E. coli* (EPEC)
EPS	Exopolysaccharide
HGT	Horizontal gene transfer
HPLC	High-performance liquid chromatography
HSL	Homoserine lactone
IBD	Inflammatory bowel disease
LAB	Lactic acid bacteria
LEE	Locus of enterocyte effacement
miRNAs	micro RNAs
MS	Mass spectrometry
NEC	Necrotising enterocolitis
OMV	Outer membrane vesicles
QQ	Quorum quenching
Qrr sRNAs	Quorum regulatory sRNAs
QS	Quorum sensing
QSM	Quorum sensing molecules
*R*-THMF	(*2R,4S*)-2-methyl-2,3,3,4-tetrahydroxytetrahydro-furan)
SAH	S-adenosylhomocysteine
SAM	S-adenosyl-L-methionine
siRNAs	Small Inhibitory RNAs
SRH	S-ribosyl-homocysteine
SSO	Specific spoilage organisms
*S*-THMF	(*2S,4S*)-2-methyl-2,3,3,4-tetra-hydroxytetrahydrofuran
TCS	Two-component systems
